# CDKN2A-Inactivated Pancreatic Ductal Adenocarcinoma Exhibits Therapeutic Sensitivity to Paclitaxel: A Bioinformatics Study

**DOI:** 10.3390/jcm9124019

**Published:** 2020-12-12

**Authors:** Jiunn-Chang Lin, Tsang-Pai Liu, Pei-Ming Yang

**Affiliations:** 1 Department of Surgery, MacKay Memorial Hospital and Mackay Medical College, Taipei 10449, Taiwan; steven4375@gmail.com (J.-C.L.); liutp@mmh.org.tw (T.-P.L.); 2 Mackay Junior College of Medicine, Nursing and Management, New Taipei City 11260, Taiwan; 3 PhD Program for Cancer Molecular Biology and Drug Discovery, College of Medical Science and Technology, Taipei Medical University, Taipei 11031, Taiwan; 4 Department of Medicine, Mackay Medical College, New Taipei City 25245, Taiwan; 5 Liver Medical Center, Mackay Memorial Hospital, Taipei 10449, Taiwan; 6 Graduate Institute of Cancer Biology and Drug Discovery, College of Medical Science and Technology, Taipei Medical University, Taipei 11031, Taiwan; 7 TMU Research Center of Cancer Translational Medicine, Taipei Medical University, Taipei 11031, Taiwan; 8 Cancer Center, Wan Fang Hospital, Taipei Medical University, Taipei 11696, Taiwan

**Keywords:** bioinformatics, CDKN2A, cell cycle, pancreatic ductal adenocarcinoma, paclitaxel

## Abstract

The mutation of cyclin dependent kinase inhibitor 2A (CDKN2A) is frequently found in pancreatic ductal adenocarcinoma (PDAC). However, its prognostic and therapeutic roles in PDAC have not been extensively investigated yet. In this study, we mined and integrated the cancer genomics and chemogenomics data to investigate the roles of CDKN2A genetic alterations in PDAC patients’ prognosis and treatment. We found that functional CDKN2A inactivation caused by mutations and deep deletions predicted poor prognosis in PDAC patients. CDKN2A inactivation was associated with the upregulation of genes related to estrogen response, which can be overcome by CDKN2A restoration. Chemosensitivity profiling of PDAC cell lines and patient-derived organoids found that CDKN2A inactivation was associated with the increased sensitivity to paclitaxel and SN-38 (the active metabolite of irinotecan). However, only paclitaxel can mimic the effect of CDKN2A restoration, and its drug sensitivity was correlated with genes related to estrogen response. Therefore, our study suggested that CDKN2A-inactivated PDAC patients could benefit from the precision treatment with paclitaxel, whose albumin-stabilized nanoparticle formulation (nab-paclitaxel) has been approved for treating PDAC.

## 1. Introduction

Pancreatic ductal adenocarcinoma (PDAC) is a cancer type with high lethality due to late diagnosis, high metastatic potential, and resistance to therapeutic agents [1,2,3,4]. The standard regimens for unresectable and advanced PDAC patients are gemcitabine-based chemotherapy, alone, or in combination with other drugs such as nab-paclitaxel (albumin-stabilized nanoparticle formulation of paclitaxel), 5-fluorouracil, irinotecan, and oxaliplatin. However, the clinical results for current regimens remain unsatisfactory [2,5]. Therefore, better therapeutic strategies are needed.

PDAC develops through the stages from low-grade dysplasia (PanIN1 and PanIN2) to high-grade dysplasia (PanIN3) [6]. The major driver gene mutation for PDAC tumorigenesis are Kirsten rat sarcoma 2 viral oncogene homolog (KRAS), tumor protein p53 (TP53), SMAD family member 4 (SMAD4), and cyclin dependent kinase inhibitor 2A (CDKN2A) [6,7]. The most common initiating mutation is the proto-oncogene KRAS. An additional mutation, such as CDKN2A, is required for the progression from PanIN1 to PanIN2. At the stage of PanIN3, inactivation of several tumor suppressor genes (TP53 and SMAD4) is observed [6].

CDKN2A is a tumor suppressor gene that encodes the p16^INK4A^ protein (hereafter mentioned as CDKN2A). As it names, CDKN2A is a negative regulator of cell cycle progression (G1-to-S phase transition) by disturbing the complex formation between CDK4/6 and cyclin D [8,9]. By using alternative open reading frames, CDKN2A also encodes p14^ARF^ protein that interacts with mouse double minute 2 homolog (MDM2) and inhibits MDM2’s ability to inhibit p53 [8,9]. CDKN2A is frequently inactivated in cancers due to genetic alterations by point mutation, homozygous deletion, promoter hypermethylation, and loss of heterozygosity [9].

In this study, we performed a bioinformatics analysis to investigate the prognostic and therapeutic impacts of CDKN2A inactivation in PDAC. We identified that paclitaxel (a microtubule inhibitor) is a potential therapeutic agent for CDKN2A-inactivated PDAC. Because nab-paclitaxel plus gemcitabine has been approved for treating metastatic pancreatic cancer [10], our results may support the usage of CDKN2A genetic status as a biomarker for precision treatment of PDAC patients by nab-paclitaxel.

## 2. Experimental Section

### 2.1. The Cancer Genome Atlas (TCGA) Data Analysis

The genetic (mutation, copy number variation, and mRNA expression) and prognostic (overall survival) data for cancer patients (“TCGA, PanCancer Atlas” data set) were obtained from the cBioPortal (accessed on 27 November 2020) [11,12]. The primary therapy outcomes for these patients were obtained from the Xena Functional Genomics Explorer (accessed on 27 November 2020) [13] by selecting a cancer type and searching the phenotype “primary_therapy_outcome_success”. The primary therapy outcomes (from better to worse) included complete remission/response (CR), partial remission/response (PR), stable disease (SD), persistent disease, and progressive disease (PD). Persistent disease, if any case existed, was classified as PD when performing analysis. The raw data were merged according to the sample ID (Table S1).

### 2.2. Gene Set Enrichment Analysis (GSEA)

Gene expression profiles for 168 PDAC patients were obtained from the cBioPortal (accessed on 27 November 2020) database [11,12]. Patients were categorized into two groups (WT + LOSS and MUT + DEEP LOSS), as described in Table S1. A microarray data set (GSE22334 [14]) was obtained from the Gene Expression Omnibus (GEO) database [15]. In this data set, a CDKN2A-null CAPAN-1 pancreatic cell line was transfected with a CDKN2A-encoding plasmid to restore its function [14]). The above gene expression data sets were subjected to gene set enrichment analysis (GSEA) against 50 cancer hallmarks [16] using the GSEA software (version 4.1.0, Broad Institute, Boston, MA, USA) [17,18].

### 2.3. Chemosensitivity Profiling in PDAC Cell Lines and Patient-Derived Organoids

The correlation between gene expression and copy numbers and chemosensitivity in PDAC cancer cell lines was analyzed using the CellMinerCDB (accessed on 27 November 2020) web-based tool [19]. The Cancer Therapeutics Response Portal (CTRP [20,21,22]) data in PDAC cell lines were used for analysis (accessed on 27 November 2020). The chemosensitivity data in PDAC patient-derived organoids with different CDKN2A genetic status were obtained from the supplementary materials of a previous study [23]. The relevant data used in this study are shown in Table S2.

### 2.4. Connectivity Map (CMap) Analysis

To identify the potential drugs being able to mimic CDKN2A activation, the differentially expressed genes (DEGs) from CDKN2-overexpressing CAPAN-1 cells (GSE22334 [14]) were prepared using the R-based web application, GEO2R [15]. These DEGs (Table S3) were queried using the Connectivity Map (CMap) database (accessed on 29 July 2020) [24]. The CMap database collects drug-induced gene expression profiles from human cancer cell lines, which can be used to compare the similarity and dissimilarity between the inputted DEGs and drug-induced gene expression [24]. Because 150 upregulated and downregulated genes were the technical limits for CMap query, only absolute fold changes (Log_2_ ratio) greater than 6.5 (Table S3; highlighted in red) were used for CMap analysis.

### 2.5. Cancer Hallmark Enrichment by the WebGeStalt Web-Based Tool

The genes positively (sensitive factors) and negatively (resistant factors) correlated with paclitaxel and SN-38 drug sensitivities in PDAC cell lines (CTRP data [20,21,22]) were obtained from the CellMinerCDB website (accessed on 03 November 2020) [19]. These sensitive and resistant factors for paclitaxel and SN-38 (Table S4) were analyzed using the WebGestalt (accessed on 03 November 2020) [25] web-based tools for cancer hallmark enrichment.

## 3. Results and Discussion

### 3.1. CDKN2A Inactivation Predicts a Poorer Prognosis in PDAC Patients

**Figure 1:**
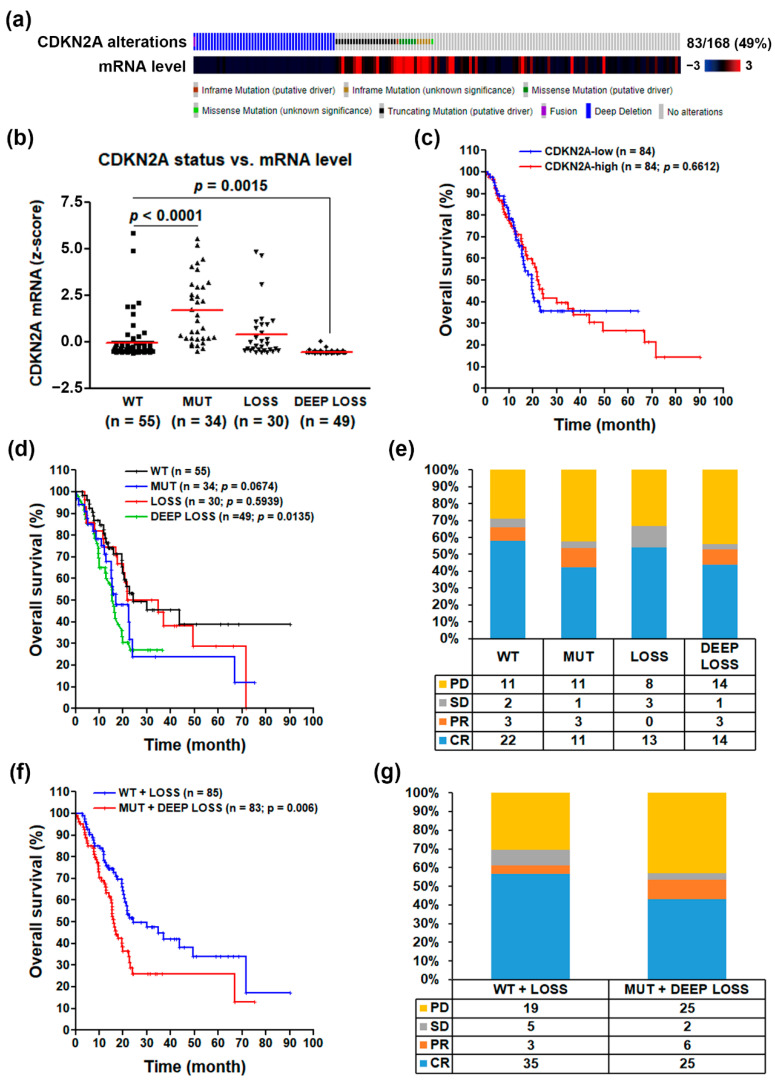
The clinical impact for the loss of cyclin dependent kinase inhibitor 2A (CDKN2A) functional activity in pancreatic ductal adenocarcinoma. (**a**) The genetic alterations and mRNA expression levels of CDKN2A gene in 168 pancreatic ductal adenocarcinoma (PDAC) patients; (**b**) the correlation between CDKN2A genetic alterations (WT for wildtype; MUT for mutation; LOSS for shallow deletion; and DEEP LOSS for deep deletion) and CDKN2A mRNA expression levels; (**c**) the correlation between CDKN2A mRNA levels and PDAC patients’ overall survivals; (**d**) the correlation between CDKN2A genetic alterations (WT; MUT; LOSS; and DEEP LOSS) and PDAC patients’ overall survivals; (**e**) the correlation between CDKN2A genetic alterations and PDAC patients’ primary therapy outcomes (PD for progressive disease; SD for stable disease; PR for partial remission/response; and CR for complete remission/response); (**f**) the correlation between CDKN2A genetic alterations (MUT + DEEP LOSS vs. WT + LOSS) and PDAC patients’ overall survivals; (**g**) the correlation between CDKN2A genetic alterations (MUT + DEEP LOSS vs. WT + LOSS) and PDAC patients’ primary therapy outcomes.

**Figure 2:**
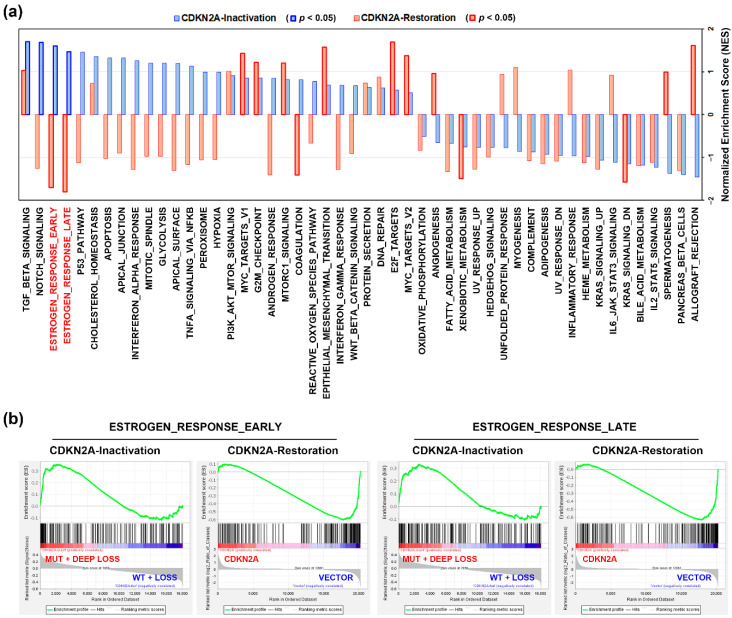
The functional impact of CDKN2A inactivation in pancreatic ductal adenocarcinoma. (**a**) Gene set enrichment analysis (GSEA) was performed against 50 cancer hallmarks. The blue bars indicate the enrichment results for CDKN2A-inactivated (“MUT + DEEP LOSS” vs. “WT + LOSS”) PDAC patients. The red bars indicate the results for CDKN2A restoration in CDKN2A-deleted PDAC cell line, CAPAN-1 (CDKN2A plasmid transfection vs. vector transfection). The blue or red bars highlighted in thick frames indicate that the cancer hallmarks were significantly enriched (*p* < 0.05); (**b**) the enrichment plots for “ESTROGEN_RESPONSE_EARLY” cancer and “ESTROGEN_RESPONSE_LATE” hallmarks.

To investigate the role of CDKN2A inactivation in PDAC, we mined the TCGA’s pancreatic adenocarcinoma (PAAD) cancer genomics data via the cBioPortal website [11,12]. We obtained 168 PDAC patients with complete genomics data (mutations, copy number variations, and mRNA levels). As shown in Figure 1a, 29% and 21% of PDAC patients harbored deep deletions and mutations of the CDKN2A gene, respectively. The most frequent mutation types were truncating and missense and in-frame mutations, which tended to have higher CDKN2A mRNA levels. To compare the impact of these genetic alterations on CDKN2A mRNA expression, a scatter plot is shown in Figure 2b. According to the definition of the TCGA database, the copy number data contained the following levels: deep deletion/loss (−2; possibly a homozygous deletion), shallow deletion/loss (−1; possibly a heterozygous deletion), diploid (0), gain (1; gain of a few additional copies), and amplification (2; gain of more copies). Two CDKN2A-wildtype (WT) PDAC patients with copy number gains were classified as WT in our analysis (Table S1a). We found that deep deletion of the CDKN2A gene resulted in the significant loss of CDKN2A mRNA expression. However, shallow deletion of the CDKN2A gene expressed similar mRNA levels compared with the WT group, suggesting that a single copy of the CDKN2A gene is enough to maintain similar mRNA levels as those in CDKN2A-WT PDAC patients. Interestingly, the CDKN2A-mutant group had higher mRNA levels compared with WT and other groups. It has been suggested that the mutant CDKN2A genes may encode functionally inactivated proteins in cancer cells [26,27,28].

The above results implied that CDKN2A mRNA expression itself was not a good prognostic biomarker for PDAC due to the high transcript levels of the mutant CDKN2A gene. Indeed, a Kaplan–Meier survival plot indicated that CDKN2A mRNA expression was not associated with PDAC patients’ overall survival (Figure 1c). Consistently, a recent study showed no significant correlation between CDKN2A mRNA/protein expression and clinical status in PDAC patients [29]. Interestingly, PDAC patients with CDKN2A mutation (MUT) or deep deletion (DEEP LOSS) had poorer overall survivals compared with those with WT or shallow deletion (LOSS) of the CDKN2A gene (Figure 1d). Similarly, the primary therapy outcomes for PDAC patients with CDKN2A-MUT and CDKN2A-DEEP LOSS were worse than those with CDKN2A-WT and CDKN2A-LOSS (Figure 1e). Therefore, we hypothesized that CDKN2A functional activity may be a more reliable biomarker for PDAC patients’ prognosis. We further classified PDAC patients into two groups: functional CDKN2A group (WT + LOSS) and inactivated CDKN2A group (MUT + DEEP LOSS). As shown in Figure 1f,g, PDAC patients with inactivated CDKN2A had worse prognostic values in overall survivals and primary therapy outcomes than those with functional CDKN2A.

Because CDKN2A is frequently inactivated in cancers [9], we further investigated whether the findings in PDAC patients could also be observed in other cancer types. We performed a pan-cancer analysis for CDKN2A alterations using the “TCGA, PanCancer Atlas” data set. As shown in Figure S1, in addition to PDAC, the high alteration frequency of the CDKN2A gene was also found in glioblastoma multiforme (GBM), head and neck squamous cell carcinoma (HNSC), esophageal carcinoma (ESCA), skin cutaneous melanoma (SKCM), lung squamous cell carcinoma (LUSC), and bladder urothelial carcinoma (BLCA). The cancer genomics (mutations, copy number variations, and mRNA levels) and patients’ survival data (Table S1b–g) in these cancer types were analyzed for the role of CDKN2A. As shown in Figure S2, both shallow and deep deletion of CDKN2A gene significantly reduced CDKN2A mRNA expression. CDKN2A mutations also reduced CDKN2A gene levels in GBM and HNSC, but had no effects on the other four cancer types. These observations were dissimilar to those found in PDAC (Figure 1b). Consistent with the observations in PDAC (Figure 1c), CDKN2A mRNA levels were not associated with cancer patients’ overall survival in GBM, ESCA, SKCM, LUSC, and BLCA (Figure S3a,c–f). Interestingly, the mutations and copy number variations of the CDKN2A gene did not affect the patients’ overall survival in GBM, ESCA, SKCM, LUSC, and BLCA (Figure S4a,c–f). An exception was that HNSC patients with lower CDKN2A mRNA levels had a poorer overall survival (Figure S3b), which may be resulted from the shallow and deep deletions, and partly mutations, of the CDKN2A gene that exhibited prognostic impact on patients’ overall survival (Figure S4b). Consistently, HNSC patients with CDKN2A gene mutations and deletions had worse primary therapy outcome (Figure S5). Therefore, we conclude that the impact of CDKN2A genetic alterations is cancer type-specific.

### 3.2. An Alteration of Estrogen Response-Related Genes by CDKN2A Inactivation in PDAC Patients

To further investigate the role of CDKN2A inactivation in PDAC, the gene expression profile in PDAC patients (TCGA-PAAD data set) with CDKN2A mutation and deep deletion (compared to those with CDKN2A wildtype and shallow deletion) were analyzed by GSEA for 50 cancer hallmark enrichment. We found that 4 cancer hallmarks (TGF-BETA_SIGNALING, NOTCH_SIGNALING, ESTROGEN_RESPONSE_EARLY, and ESTROGEN_RESPONSE_LATE) were significantly associated with CDKN2A inactivation (mutations and deep deletions) in PDAC patients (Figure 2a, the blue bars). To dissect the most important cancer hallmark associated with CDKN2A inactivation, we performed GSEA using a microarray data set (GSE22334 [14]), in which a CDKN2A-deleted PDAC cell line, CAPAN-1, was transfected with a CDKN2A-encoding plasmid to restore its function [14]. As shown in Figure 2a (the red bars), CDKN2A restoration downregulated the CDKN2A-associated cancer hallmarks (ESTROGEN_RESPONSE_EARLY and ESTROGEN_RESPONSE_LATE). The enrichment plots for these two cancer hallmarks are shown in Figure 2b. Therefore, CDKN2A inactivation may alter the estrogen response in PDAC.

Because CDKN2A inactivation (LOSS + DEEP LOSS) in HNSC predicted patients’ overall survival and primary therapy outcome (Figures S4b and S5), we also performed GSEA as a comparison. As shown in Figure S6, both ESTROGEN_RESPONSE_EARLY and ESTROGEN_ RESPONSE_LATE cancer hallmarks were not significantly enriched in CDKN2A-inactivated HNSC patients. Instead, ANGIOGENESIS, UNFOLDED_PROTEIN_RESPONSE, and GLYCOLYSIS cancer hallmarks were enriched. This result further indicates that the role of CDKN2A inactivation is cancer type-specific.

### 3.3. Loss of CDKN2A Exhibits Therapeutic Sensitivity to Paclitaxel and SN-38 in PDAC

**Figure 3:**
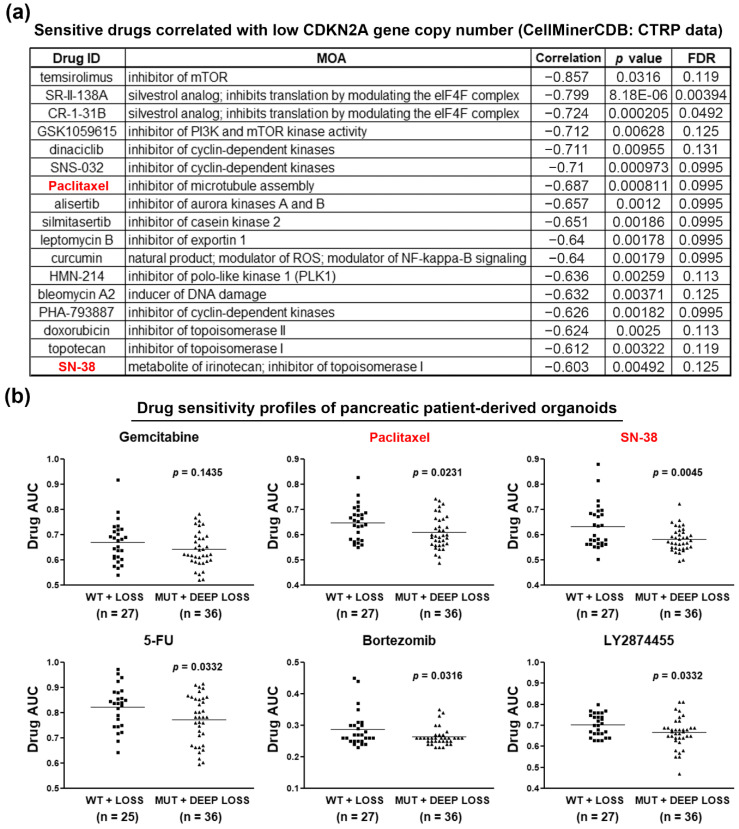
Chemosensitivity profiles in CDKN2A-inactivated PDAC cell lines and patient-derived organoids. (**a**) The correlation between CDKN2A gene copy numbers and drug sensitivities in PDAC cell lines were obtained from the CTRP data via the CellMinerCDB website. Sensitive drugs correlated with low CDKN2A gene copy numbers are shown; (**b**) drug response data in PDAC patient-derived organoids were obtained from the supplementary materials in a previous study (reference [23]). Lower area under the curve (AUC) values indicated that the organoids were more sensitive to drug treatment.

Given the fact that gemcitabine is the standard treatment for PDAC patients, the above results (Figure 1) indicate that PDAC patients with functional CDKN2A may be suitable for gemcitabine treatment. For those with inactivated CDKN2A, the development of an alternative therapeutic strategy is needed. To identify potential treatment for CDKN2A-inactivated PDAC patients, we mined the drug sensitivity profiles of PDAC cell lines via the CTRP database [20,21,22]. Because the reported CDKN2A mutation status is contradictory [30], we analyzed the correlation between drug sensitivity and CDKN2A gene copy numbers. As shown in Figure S7, PDAC cells with copy numbers <−2 (deep deletion) had low CDKN2A expression levels. The sensitive drugs for PDAC cell lines with low CDKN2A gene copy numbers are shown in Figure 3a. Because CDKN2A disturbs CDK4/6 and cyclin D complex formation and negatively regulates G1/S cell cycle progression [8,9], it was reasonable that CDKN2A-inactivated PDAC cell lines were sensitive to several CDK inhibitors (dinaciclib, SNS-032, and PHA-793887). Interestingly, CDKN2A-inactivated PDAC cells were also sensitive to cell cycle-targeting drugs, including silmitasertib (casein kinase 2 inhibitor), bleomycin A2 (DNA damage inducer), and those targeting mitosis (paclitaxel, alisertib, HMN-214) and topoisomerases (doxorubicin, topotecan, and SN-38). Therefore, CDKN2A inactivation correlates with the increased sensitivity to cell cycle-targeting drugs in PDAC cell lines.

To accelerate the clinical application of our findings, we further investigate the correlation between CDKN2A status and therapeutic effects of paclitaxel and SN-38 (the active metabolite of irinotecan), which have been approved for treating PDAC. We employed drug sensitivity profiles obtained from PDAC patient-derived organoids in a previous study [23]. The organoids were categorized into two groups (MUT + DEEP LOSS vs. WT + LOSS) and their sensitivities to chemotherapeutic and molecular-targeted drugs were compared. As shown in Table S2 and Figure 3b, CDKN2A-inactivated (MUT + DEEP LOSS) organoids were more sensitive to paclitaxel, SN-38, 5-FU, bortezomib, and LY2874455, which confirmed the in vitro effects of paclitaxel and SN-38 (Figure 3a). Therefore, we conclude that CDKN2A-inactivated PDAC patients may benefit from the treatments with clinically approved nab-paclitaxel and irinotecan.

To investigate whether CDKN2A inactivation in GBM, ESCA, SKCM, LUSC, and BLCA also led to the increased sensitivity to paclitaxel and SN-38, the correlation between CDKN2A gene copy number variation and drug sensitivity was analyzed using the data obtained from the CTRP database. As shown in Figures S8 and S9, skin melanoma (MEL) cell lines with lower copy numbers had higher sensitivity to SN-38. In contrast, the central nervous system (CNS) cancer cell lines with lower copy numbers were more resistant to both paclitaxel and SN-38. Other cancer types did not exhibit a significant correlation between CDKN2A copy numbers and drug sensitivity. Therefore, the observations from PDAC may not be applied to other cancer types with a high frequency of CDKN2A alterations.

### 3.4. Paclitaxel Treatment Mimics the Gene Expression Profile of CDKN2A Restoration

**Figure 4:**
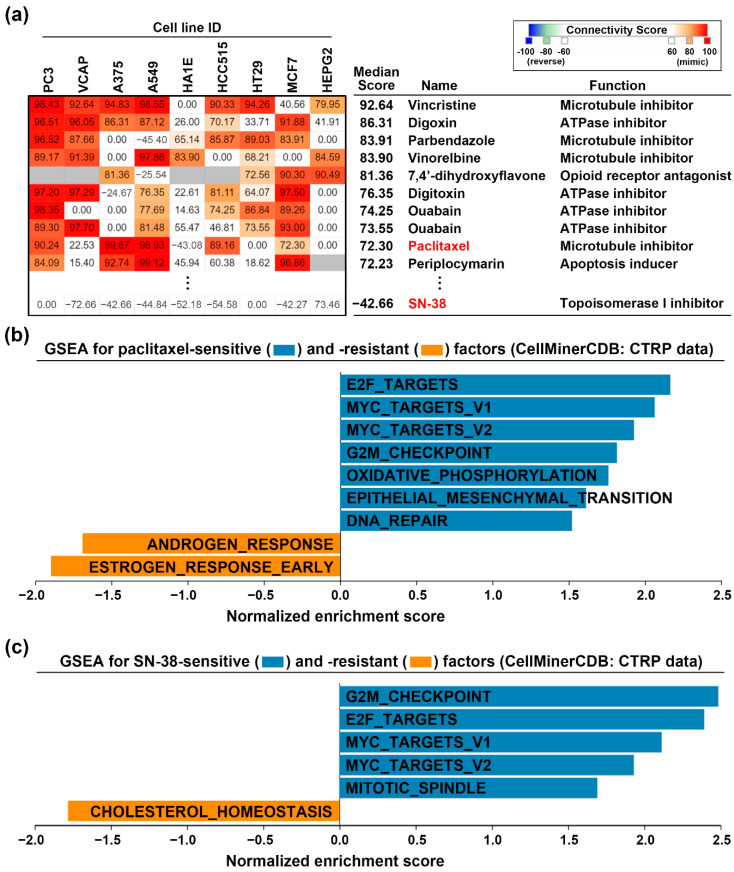
Paclitaxel treatment mimics the effect of CDKN2A restoration. (**a**) CMap analysis was performed to explore the similarity among CDKN2A- and drug-induced gene signatures; (**b**,**c**) the sensitive and resistant gene factors for paclitaxel (**b**) and SN-38 (**c**) in PDAC cell lines were obtained from the CTRP database via an online tool, the CellMinerCDB. GSEA was performed for these factors against 50 cancer hallmarks.

To investigate whether paclitaxel and SN-38 could restore CDKN2A functional activity, we employed the CMap analysis. CMap database allows users to query a gene signature and explore the connections between the queried gene signature and drug-driven gene expression [24]. We prepared the DEGs (Table S3) from CDKN2A-overexpressing CAPAN-1 cells and queried the CMap database. Figure 4a shows the top 10 perturbagens that mimicked the CDKN2A-driven gene signature, including paclitaxel with the median connectivity score of 72.3. In contrast, the median connectivity score of SN-38 is −42.66. Therefore, paclitaxel treatment may mimic the effect of CDKN2A overexpression. The above result implied that paclitaxel, but not SN-38, may reverse the CDKN2A-associated cancer hallmarks (ESTROGEN_RESPONSE_EARLY and ESTROGEN_RESPONSE_LATE). To confirm this hypothesis, we obtained the genes whose expression levels were positively (sensitive factors) and negatively (resistant factors) correlated with the drug activity of paclitaxel and SN-38 (Table S4). These sensitive and resistant factors were analyzed by GSEA for cancer hallmark enrichment. As shown in Figure 4b,c, the cancer hallmark “ESTROGEN_RESPONSE_EARLY” was negatively correlated with paclitaxel, but not SN-38, activity in PDAC cell lines. Therefore, paclitaxel treatment can mimic the gene expression profile of CDKN2A restoration.

## 4. Conclusions

This study integrates publicly available data to investigate the role of the CDKN2A gene in PDAC. The results suggest that the CDKN2A functional inactivation caused by mutations and deep deletions predicts poor prognosis in PDAC patients. Besides, CDKN2A inactivation results in the upregulation of estrogen response-related genes, which can be reversed by paclitaxel. Our study provides a basis for the precision treatment of CDKN2A-inactivated PDAC patients by paclitaxel.

## Supplementary Materials

The following are available online at https://www.mdpi.com/2077-0383/9/12/4019/s1, Table S1: Cancer genomics data for cancer patients, Table S2: Chemosensitivity profiles of patient-derived PDAC organoids, Table S3. Differentially expressed genes in CDKN2A-overexpressed CAPAN-1 cells, Table S4. Genes correlated with paclitaxel and SN-38 drug sensitivity in PDAC cell lines, Figure S1: A pan-cancer analysis for the CDKN2A genetic alterations, Figure S2. Effect of genetic alterations on CDKN2A mRNA expression in cancers, Figure S3. Effect of CDKN2A mRNA expression levels on cancer patients’ overall survival, Figure S4. Effect of CDKN2A genetic alterations on cancer patients’ overall survival, Figure S5. Effect of CDKN2A genetic alterations on cancer patients’ primary therapy outcome, Figure S6. The functional impact of CDKN2A inactivation in head and neck squamous cell carcinoma, Figure S7. The correlation between CDKN2A copy number variation and mRNA expression levels in pancreatic ductal adenocarcinoma cell lines, Figure S8. The correlation between CDKN2A copy number variation and paclitaxel drug activity in cancer cells, Figure S9. The correlation between CDKN2A copy number variation and SN-38 drug activity in cancer cells.
