# Supplementary material for: CDKN2A-Inactivated Pancreatic Ductal Adenocarcinoma Exhibits Therapeutic Sensitivity to Paclitaxel: A Bioinformatics Study
Source: J Clin Med. 2020 Dec 12;9(12):4019. doi: 10.3390/jcm9124019 (PMC7763913; doi:10.3390/jcm9124019)
Supplement: Supplementary file 1 [file jcm-09-04019-s001.zip › jcm-1006906supple/Supplementary Figures.pdf]

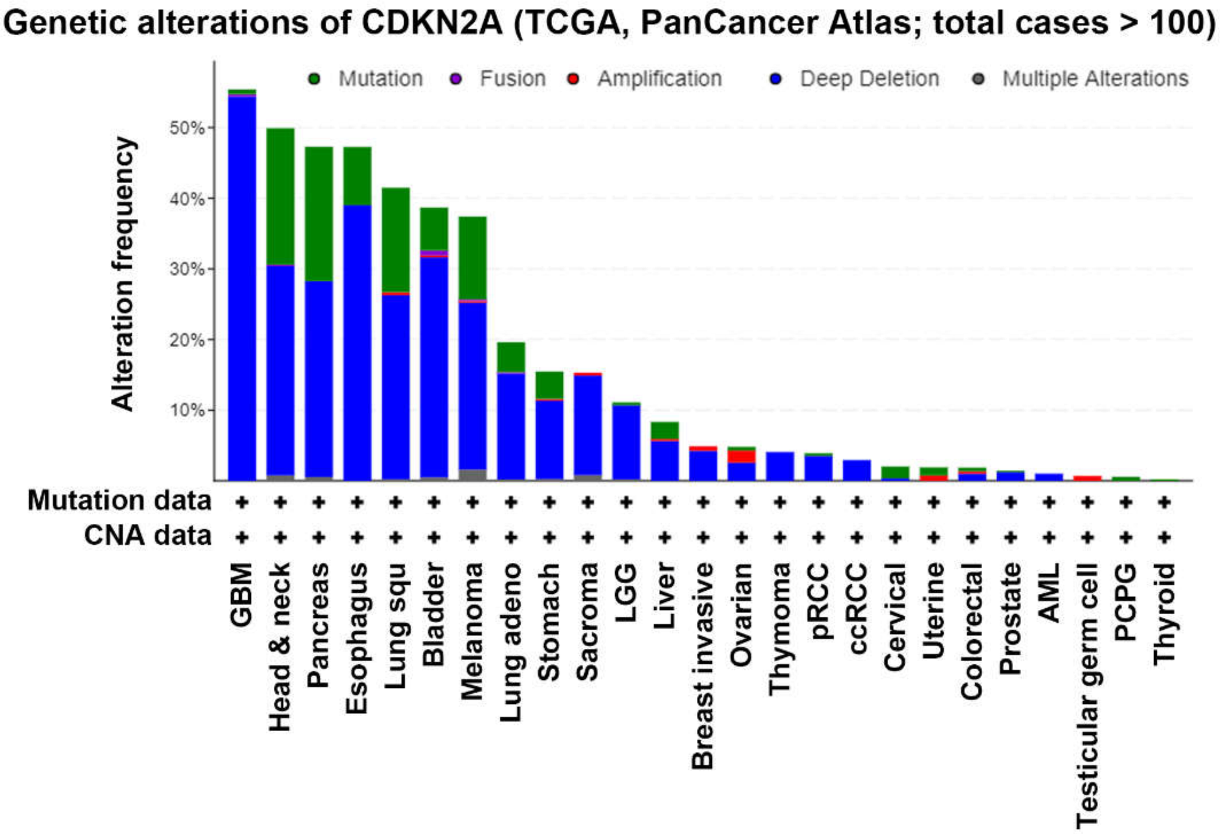

Figure S1. A pan-cancer analysis for the CDKN2A genetic alterations. CDKN2A gene was analyzed for mutation status and copy number alterations in various cancer types using "TCGA, PanCancer Atlas" data set in the cBioPortal cancer genomics database. The studies with total cases less than 100 were excluded.

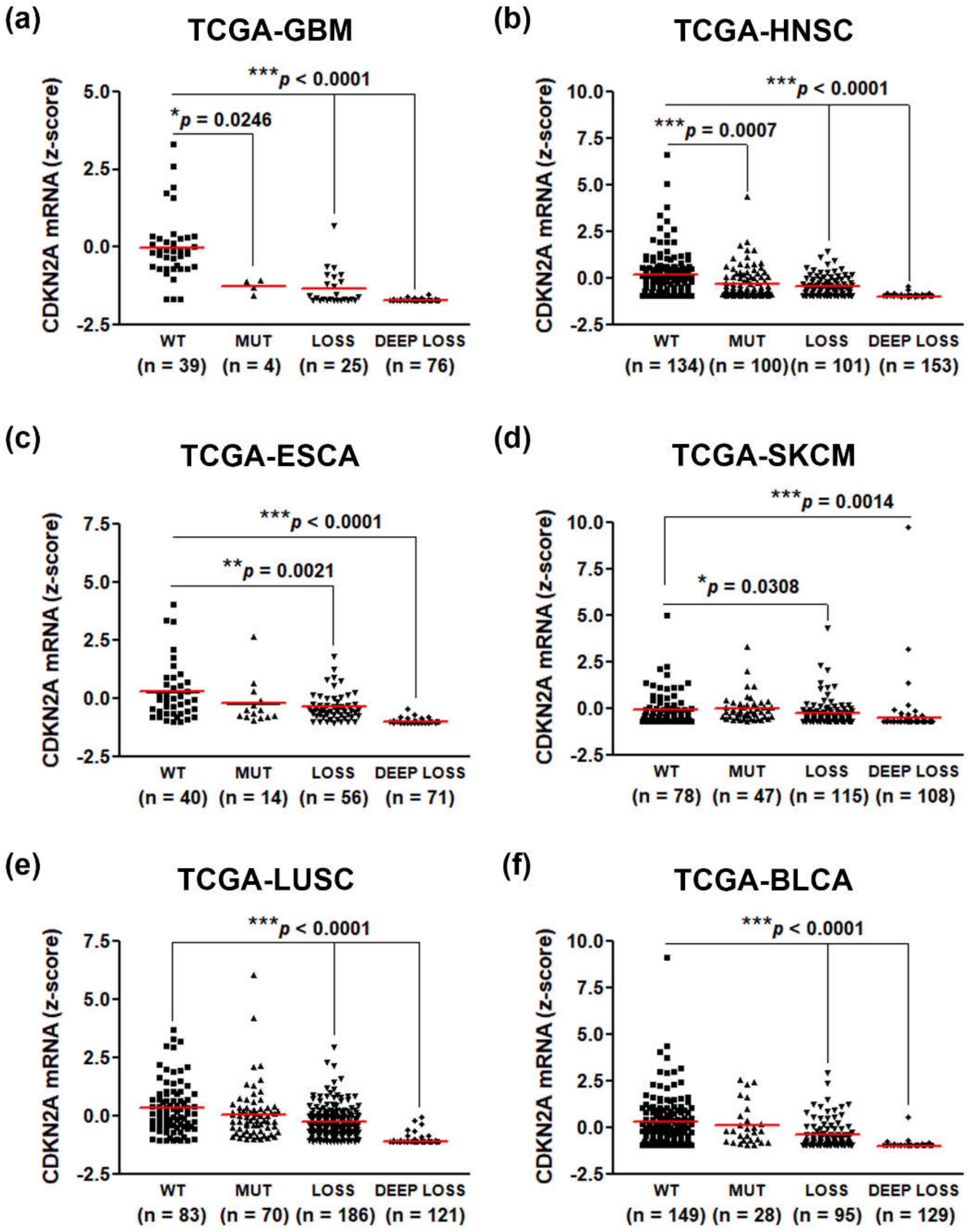

Figure S2. Effect of genetic alterations on CDKN2A mRNA expression in cancers. The genetic alterations and mRNA expression levels of CDKN2A gene in GBM **(a)**, HNSC **(b)**, ESCA **(c)**, SKCM **(d)**, LUSC **(e)**, and BLCA **(f)** were obtained from the cBioPortal cancer genomics database. "TCGA, PanCancer Atlas" data set was used for analysis.

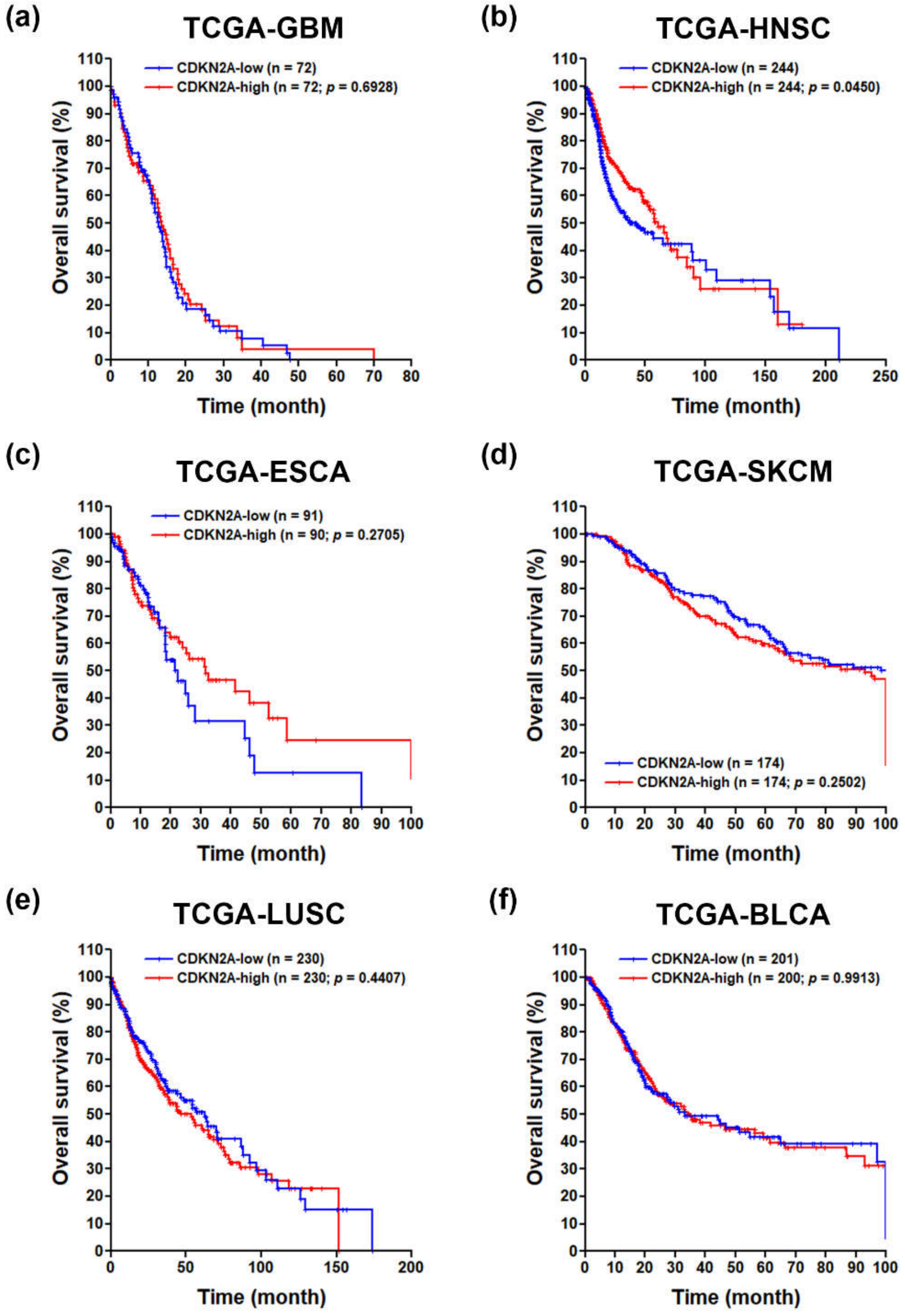

Figure S3. Effect of CDKN2A mRNA expression levels on cancer patients' overall survival. The CDKN2A mRNA expression levels and overall survival data in GBM **(a)**, HNSC **(b)**, ESCA **(c)**, SKCM **(d)**, LUSC **(e)**, and BLCA **(f)** were obtained from the cBioPortal cancer genomics database. "TCGA, PanCancer Atlas" data set was used for analysis.

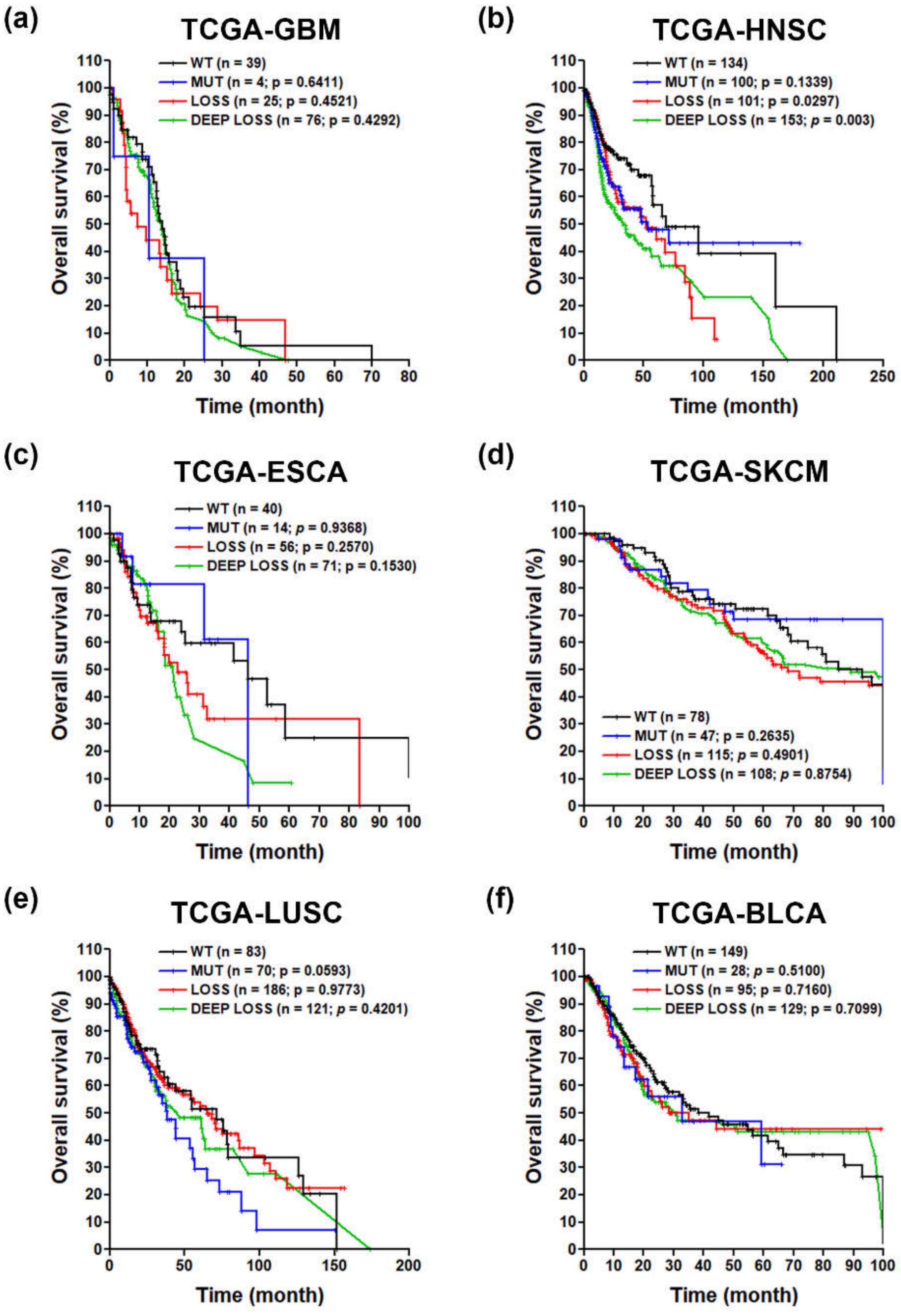

Figure S4. Effect of CDKN2A genetic alterations on cancer patients' overall survival. The CDKN2A genetic (WT for wildtype; MUT for mutation; LOSS for shallow deletion; and DEEP LOSS for deep deletion) and overall survival data in GBM **(a)**, HNSC **(b)**, ESCA **(c)**, SKCM **(d)**, LUSC **(e)**, and BLCA **(f)** were obtained from the cBioPortal cancer genomics database. "TCGA, PanCancer Atlas" data set was used for analysis.

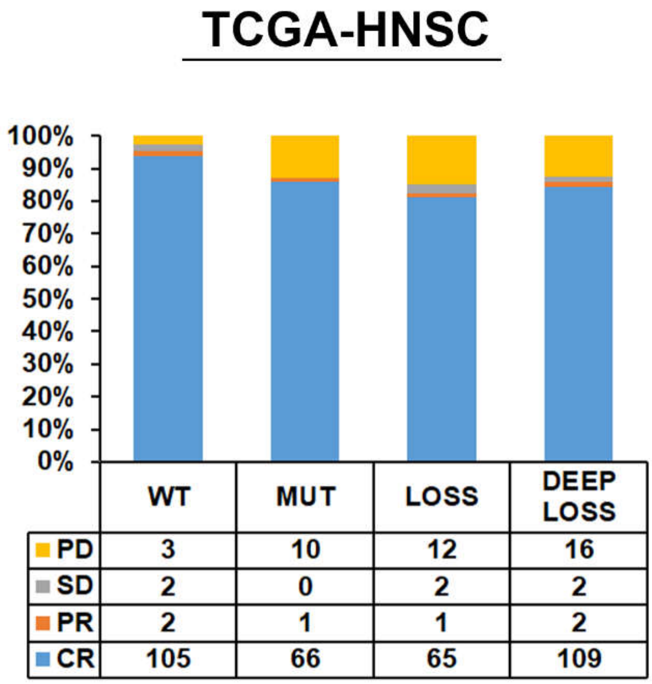

Figure S5. Effect of CDKN2A genetic alterations on cancer patients' primary therapy outcome. The CDKN2A genetic data (WT for wildtype; MUT for mutation; LOSS for shallow deletion; and DEEP LOSS for deep deletion) in HNSC were obtained from the cBioPortal cancer genomics database. "TCGA, PanCancer Atlas" data set was used for analysis. HNSC patients' primary therapy outcome data (PD for persistent and progressive disease; SD for stable disease; PR for partial remission/response; and CR for complete remission/response) were obtained from the Xena Functional Genomics Explorer.

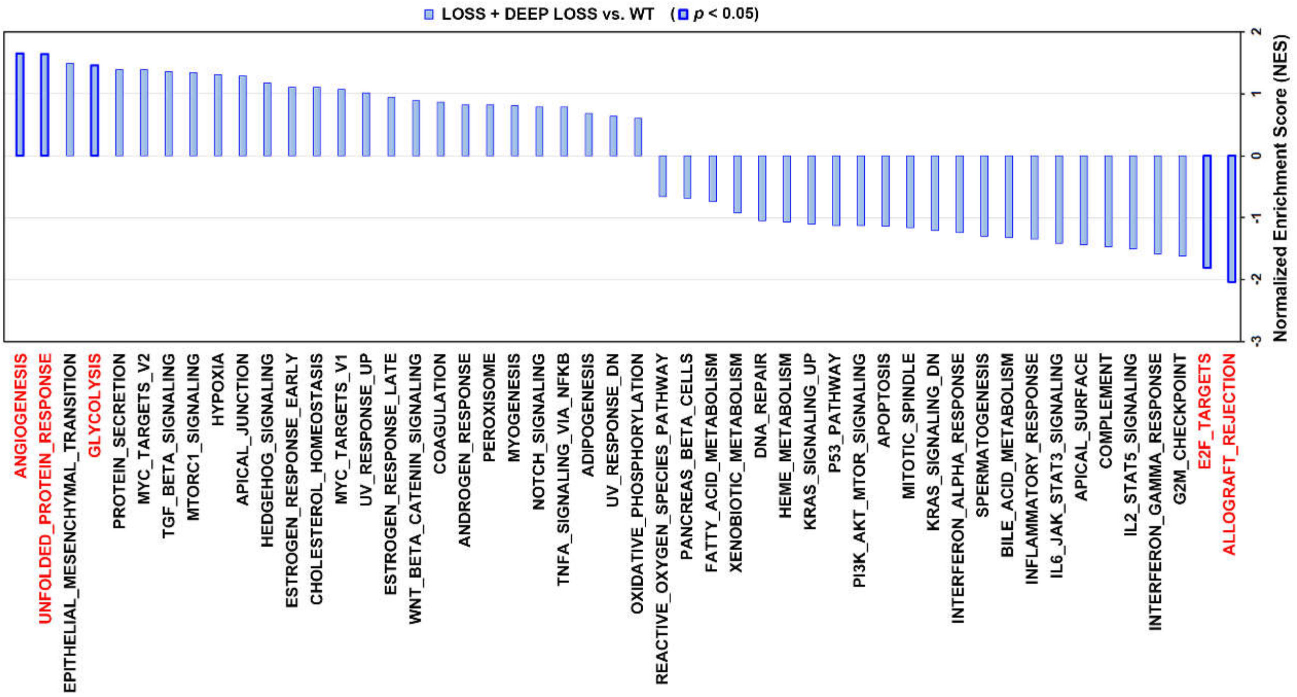

Figure S6. The functional impact of CDKN2A inactivation in head and neck squamous cell carcinoma. Gene set enrichment analysis (GSEA) was performed against 50 cancer hallmark enrichment for CDKN2A-inactivated (LOSS + DEEP LOSS vs. WT) HNSC patients. The blue bars highlighted in thick frames indicated that the cancer hallmarks (highlighted in red) were significantly enriched ($p < 0.05$).

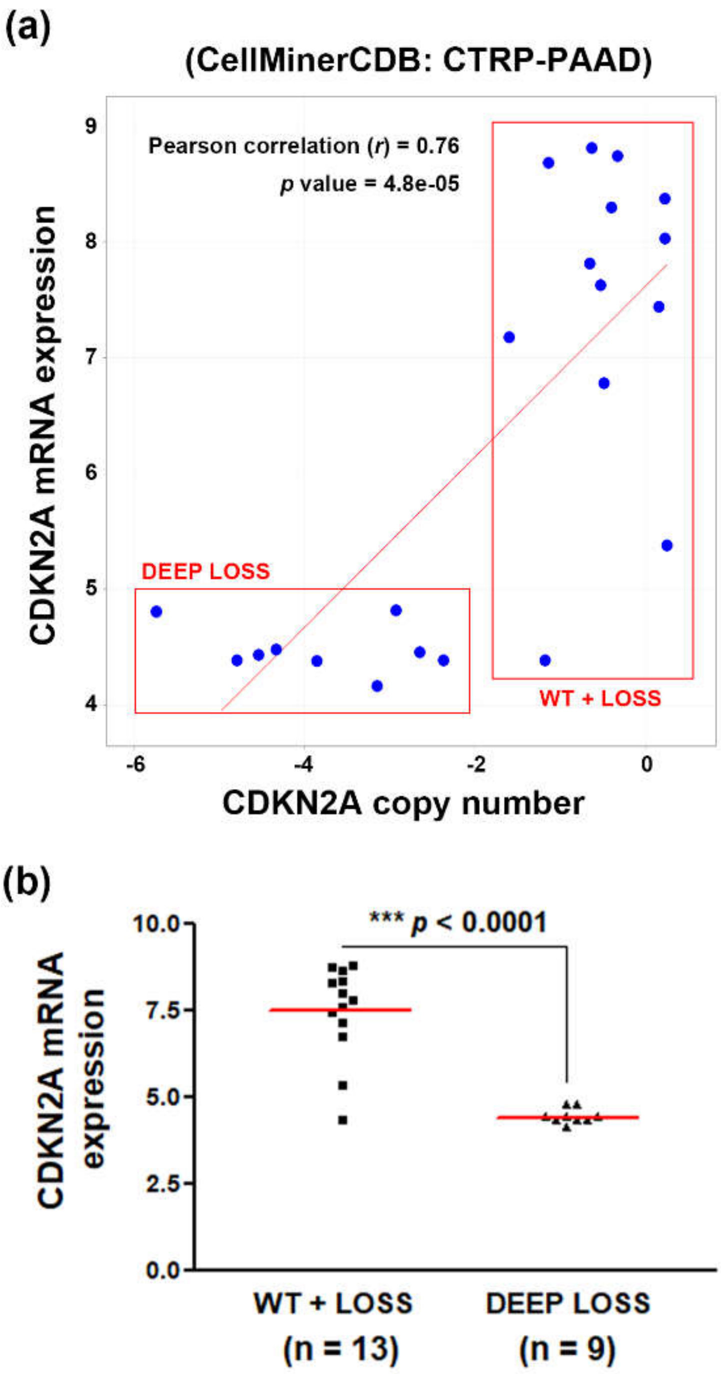

Figure S7. The correlation between CDKN2A copy number variation and mRNA expression levels in pancreatic ductal adenocarcinoma cell lines. (**a**) The CDKN2A gene copy numbers and mRNA expression levels were obtained from the CTRP database via an online tool, the CellMinerCDB. The cell lines (blue dots) were grouped into two categories: “WT + LOSS” and “DEEP LOSS”. **(b)** The difference between “WT + LOSS” and “DEEP LOSS” was calculated by a nonparametric *t*-test.

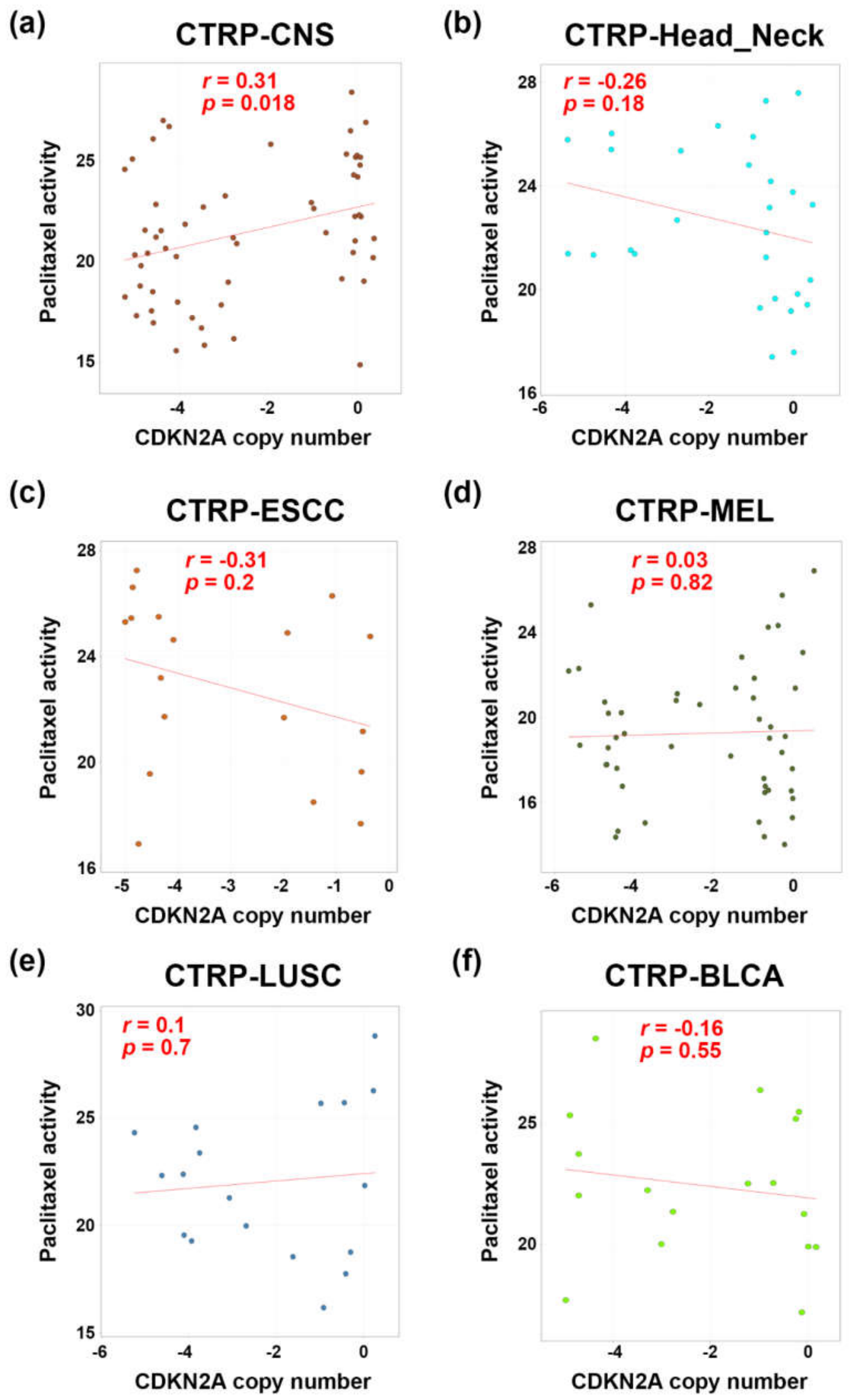

Figure S8. The correlation between CDKN2A copy number variation and paclitaxel drug activity in cancer cells. The CDKN2A gene copy numbers and paclitaxel drug activities in cell lines derived from the central nervous system (CNS) **(a)**, head and neck **(b)**, esophageal squamous cell carcinoma (ESCC) **(c)**, skin melanoma (MEL) **(d)**, lung squamous cell carcinoma (LUSC) **(e)**, and bladder urothelial carcinoma (BLCA) **(f)** were obtained from the CTRP database via an online tool, the CellMinerCDB.

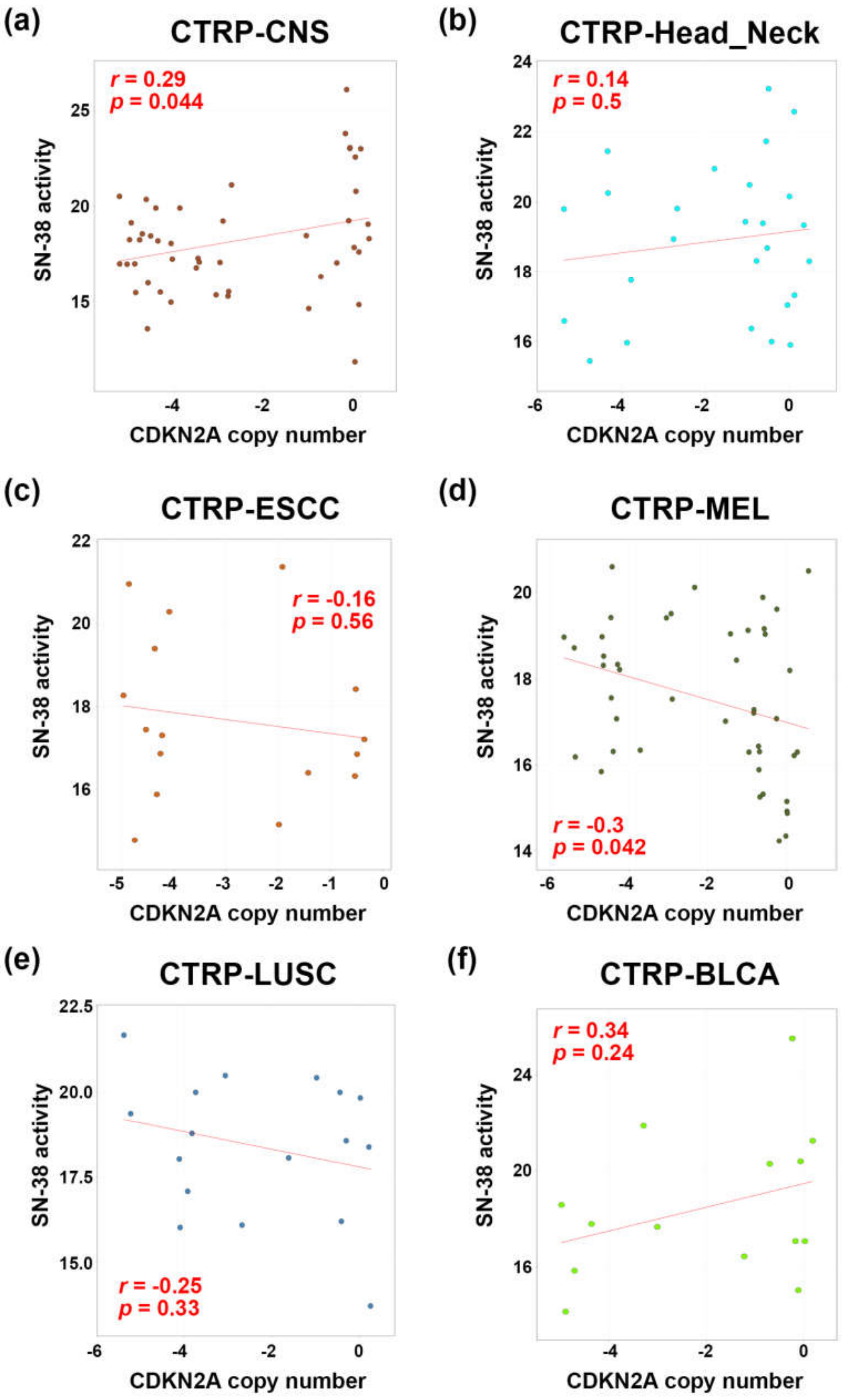

Figure S9. The correlation between CDKN2A copy number variation and SN-38 drug activity in cancer cells. The CDKN2A gene copy numbers and SN-38 drug activities in cell lines derived from the central nervous system (CNS) **(a)**, head and neck **(b)**, esophageal squamous cell carcinoma (ESCC) **(c)**, skin melanoma (MEL) **(d)**, lung squamous cell carcinoma (LUSC) **(e)**, and bladder urothelial carcinoma (BLCA) **(f)** were obtained from the CTRP database via an online tool, the CellMinerCDB.
